# Interleukin-10 Mitigates Doxorubicin-Induced Endoplasmic Reticulum Stress as Well as Cardiomyopathy

**DOI:** 10.3390/biomedicines10040890

**Published:** 2022-04-13

**Authors:** Akshi Malik, Ashim K. Bagchi, Davinder S. Jassal, Pawan K. Singal

**Affiliations:** 1 Department of Physiology and Pathophysiology, Institute of Cardiovascular Sciences, St. Boniface Hospital Albrechtsen Research Centre, University of Manitoba, Winnipeg, MB R2H 2A6, Canada; amalik@sbrc.ca (A.M.); djassal@sbgh.mb.ca (D.S.J.); 2 Department of Internal Medicine, Cardiology Division, Central Arkansas Veterans Healthcare System, University of Arkansas for Medical Sciences, Little Rock, AR 72205, USA; abagchi@uams.edu; 3 Section of Cardiology, Rady Faculty of Health Sciences, Max Rady College of Medicine, University of Manitoba, Winnipeg, MB R2H 2A6, Canada

**Keywords:** doxorubicin-induced cardiomyopathy, interleukin-10, activating transcription factor 6α, endoplasmic reticulum stress-induced apoptosis

## Abstract

The use of doxorubicin (Dox) in cancer patients carries the risk of cardiotoxicity via an increase in oxidative stress, mitochondrial dysfunction, and disturbed endoplasmic reticulum (ER) homeostasis in cardiomyocytes. The present study explores which of the ER transmembrane sensors is involved in Dox-induced apoptosis and whether interleukin-10 (IL-10) has any mitigating effect. There was a time-related increase in apoptosis in cardiomyocytes exposed to 5.43 µg/mL Dox for 0 to 48 h. Dox treatment for 24 h significantly upregulated glucose-regulated proteins 78 and 94, protein disulfide isomerase, cleavage of activating transcription factor 6α, and X-box binding protein 1. These Dox-induced changes in ER stress proteins as well as apoptosis were blunted by IL-10 (10 ng/mL). In Dox-exposed cardiomyocytes, IL-10 also promoted expression of protein kinase-like endoplasmic reticulum kinase and inositol-requiring kinase 1α, which helped in maintaining ER homeostasis. Additionally, under Dox-treatment, IL-10 downregulated caspase-12 activation as well as phosphorylation of c-JUN NH2-terminal kinase, thereby promoting cardiomyocyte survival. IL-10 was able to reduce the overexpression of mitochondrial apoptotic proteins caspase-3 as well as Bax, which were upregulated upon Dox treatment. Thus, a reduction in Dox-induced ER stress as well as apoptosis through IL-10 may provide a significant benefit in improving cardiac function.

## 1. Introduction

Since its discovery in the 1960s, doxorubicin (Dox) has been widely used as a chemotherapeutic drug for several types of cancers such as breast, ovary, bladder, thyroid, leukemias, soft-tissue sarcomas, and solid tumors [1,2]. However, the long-term use of Dox is limited due to the risk of developing significant cardiotoxicity in some patients [3,4]. Despite decades of research, there are still gaps in our understanding of Dox-induced cardiomyopathy (DIC) and heart failure.

The subcellular basis of DIC is multifactorial but an increase in oxidative stress (OS) appears to be one of the key factors [4,5]. Dox-mediated redox cycling and generation of reactive oxygen species (ROS) is known to activate signaling precursors leading to DNA damage, mitochondrial dysfunction, attenuation in protein synthesis, and disturbed intracellular calcium homeostasis [6,7,8]. In addition to functional changes, Dox can also induce subcellular changes such as vacuolization of the cytoplasm and dilation of endoplasmic reticulum (ER) [3,4]. The ER is important in the maintenance of excitation–contraction coupling, calcium homeostasis, protein synthesis, protein folding, and protein translocation [9,10]. Stress and/or pathological stimuli such as disrupted redox cycling can affect ER homeostasis referred to as ER stress, which results in the accumulation of unfolded and/or misfolded proteins. This accumulation of unfolded proteins in the ER leads to the unfolded protein response (UPR) [11,12], through the activation of protein kinase-like endoplasmic reticulum kinase (PERK), activating transcription factor 6α (ATF6α) and inositol-requiring kinase 1α (IRE1α) [8,13]. Under stress conditions, luminal ER chaperone, glucose-regulated protein 78 (GRP78) is released and allows dimerization and auto-phosphorylation of PERK as well as IRE1α, and translocation of ATF6 for its cleavage and further processing in the nucleus [14]. PERK is responsible for the attenuation of protein translation through phosphorylation of eukaryotic translation initiation factor-2α (eIF-2α), which in turn leads to the translation of activating transcription factor 4 (ATF4) to induce an antioxidant response and an increase in ER protein folding capacity [15,16].

IRE1α is the most conserved arm of the UPR and is responsible for the upregulation of ER-associated degradation (ERAD) of misfolded/unfolded proteins via the ERAD complex, as well as upregulating anti-inflammatory cytokines, antioxidant responses, and ER chaperones [17]. IRE1α also induces unconventional splicing of X-box binding protein 1 (XBP1) mRNA. The splicing of XBP1 mRNA generates a highly active transcription factor: spliced XBP1 (XBP1s) and unspliced XBP1 (Xbp1u). XBP1s translocates to the nucleus for the subsequent upregulation of transcription factors involved in ERAD, lipid biogenesis, inflammation, and autophagy [18,19]. On the other hand, proteolysis of ATF6 and further processing in the Golgi releases an active form of ATF6 to the nucleus. Cleaved-ATF6 further induces the expression of XBP1 mRNA and chaperones such as glucose-regulated protein 94 (GRP94), GRP78, and/or protein disulfide isomerase (PDI), and thus plays a major role in adaptive responses to ER stress [19,20]. However, if the UPR fails to restore ER homeostasis, ER stress-initiated apoptotic signaling pathways are activated via CCAAT/enhancer homologous protein (CHOP) [21], caspase-12 activation [22,23,24], phosphorylation of c-JUN NH_2_-terminal kinase (JNK) [17], and pro-apoptotic gene expression, which all play important roles in cell dysfunction and apoptosis [8,25].

Several natural and chemical compounds that could improve Dox-induced ER stress in heart tissues have been reviewed [26]. However, we and others have recently reported that interleukin-10 (IL-10) can mitigate OS as well as inflammatory responses under ischemia–reperfusion [27,28], attenuate pressure-overload injury [29], and improve cardiac function [30,31]. In the present study on isolated cardiomyocytes, we examined Dox-induced ER stress, and ER signaling events responsible for the activation of DIC and their mitigation by IL-10.

## 2. Materials and Methods

### 2.1. Cardiomyocyte Isolation and Treatment

Cardiomyocytes were isolated from male Sprague-Dawley rat hearts (200–250 g) using standard Langendorff apparatus procedures as previously described [8] and were plated (10^6^ per dish) in 10 cm laminin-coated (20 μg/mL) polystyrene tissue culture dishes (Corning, #CLS430167, Millipore Sigma, Oakville, ON, Canada). Cells were incubated at 37 °C in 5% CO_2_-95% O_2_ in M199 (Sigma, Rockville, MD, USA) culture media supplemented with 10% fetal bovine serum (FBS) (Life Technologies, Carlsbad, CA, USA) and streptomycin/penicillin (100 U). Unattached dead cells were removed after 2 h by washing with M199 culture media and viable cardiomyocytes were incubated overnight in M199 with 0.5% FBS under the same incubation conditions. Cardiomyocytes were divided into four treatment groups as follows: control (cardiomyocytes cultured in M199 + 0.5% FBS); IL-10 (10 ng/mL) (R&D Systems, #547-RL-010, Minneapolis, MN, USA); Dox (5.43 µg/mL) (Doxorubicin hydrochloride 10 mg/mL, Pfizer Canada, Kirkland, QC, Canada); and Dox + IL-10, for 4, 12, and 24 h. For the combination group of Dox + IL-10, cells were treated with IL-10 for 1 h before the addition of Dox.2.2. Cell Viability

To determine viability of cardiomyocytes in different treatment groups at different time points (0–48 h), MTT (3-(4,5-dimethylthiazol-2-yl)-2,5-diphenyltetrazolium bromide), a tetrazole, was used. After the treatment period, cardiomyocytes (10^4^ cells/well) plated on 96-well plates were incubated with 5 mg/mL MTT at 37 °C for 2 h. The supernatant was removed carefully and 150 μL of dimethyl sulfoxide was added to each well and mixed thoroughly using a pipette to dissolve the formazan crystals formed. The absorbance of each well was recorded at 570 nm using a microwell plate reader (Cytation 5, BioTek Agilent, Santa Clara, CA, USA). Cell population was also examined under phase contrast microscope to determine the number of rod-shaped cardiomyocytes (Cytation 5, BioTek Agilent, Santa Clara, CA, USA).

### 2.2. Protein Estimation

Cardiomyocytes from different treatment groups were washed with PBS and were centrifuged at 3000 rpm for 10 min. The pellet was resuspended overnight in an ice-cold cell lysis RIPA buffer containing protease inhibitor cocktail (Roche Diagnostics, Mississauga, ON, Canada), phosphatase inhibitor (Pierce-Thermo-Fisher Scientific, Waltham, MA, USA), 1 mM phenylmethylsulfonyl fluoride, and DTT and then sonicated the next day using a water bath sonicator. Sonicated samples were then centrifuged at 12,000 rpm for 20 min at 4 °C. The supernatant was collected in separate tubes and stored at −20 °C for further analysis. Total protein concentration of all samples was determined using BSA as standard according to the modified Bio-Rad procedure using a microwell plate reader (Cytation 5, BioTek, Agilent, Santa Clara, CA, USA).

### 2.3. Western Blot Analysis

Protein, 20–30 µg, was separated by electrophoresis on 10–12% SDS-polyacrylamide gels at 60–100 mV and then transferred onto polyvinylidene fluoride (PVDF) membranes for 90 min on constant 200 mA at 4 °C. After protein transfer, membranes were blocked in 5% BSA + Tris-buffered saline plus Tween-20 (TBST) blocking buffer for an hour, then incubated with the respective primary antibodies overnight at 4 °C. Following 3 washes with TBST, the membranes were incubated with horseradish peroxidase (HRP)-conjugated anti-rabbit IgG (Promega,#W401B, Madison, WI, USA) or HRP-conjugated anti-mouse IgG secondary antibodies (BioRad, #170-6516, Mississauga, ON, Canada) (1:5000) for 1 h at room temperature. Signals were detected using ECL plus kit reagents (Perkin Elmer, Guelph, ON, Canada) on a Chem Doc imager (BioRad, Mississauga, ON, Canada). Primary antibodies used were: Anti-Caspase-12 (Abcam, #ab62484, Toronto, ON, Canada); Anti-KDEL (Abcam, #ab176333, Toronto, ON, Canada), which recognizes GRP94, GRP78, and PDI; Anti-XBP1(Abcam, #ab37152, Toronto, ON, Canada); Anti-ATF6 (Proteintech, #24169-1-AP, Rosemont, IL, USA); Anti-PERK (Proteintech, #20582-1-AP, Rosemont, IL, USA); Anti-IRE1α (Proteintech, #27582-1-AP, Rosemont, IL, USA).

### 2.4. Immunofluorescence

Cardiomyocytes were fixed with 4% paraformaldehyde (PFA) for 10 min at RT in 8-chambered slides and were then washed with PBS-Tween 20 (PBS-T) twice. Non-specific sites were blocked by incubating in blocking buffer (5% BSA + 0.1% Triton X in PBS) for 1 h and washing twice with washing buffer (10% blocking buffer in 1X PBS). ATF6 primary antibody incubation (1:100) was conducted overnight at 4 °C, followed by two washes. Alexa Fluor 647 (Red) (1:250) secondary antibody (Invitrogen, #A21246, Life Technologies, Eugene, OR, USA) incubation was conducted for an hour in the dark at RT. After three washes, slides were mounted in DAPI containing mounting media (Millipore Sigma, Oakville, ON, Canada) and images were acquired on a Nikon Eclipse Ti2 microscope.

### 2.5. RT-qPCR

Total RNA was extracted using Aurum™ Total RNA Mini Kit (BioRad, #7326820, Hercules, CA, USA) according to the manufacturer’s instructions. RNA quantification was performed using a NanoDrop (NanoDrop Lite, Thermo Scientific, Waltham, MA, USA). Total RNA (40 ng) from each sample was used for cDNA synthesis using the High-Capacity cDNA Reverse Transcription Kit with RNase Inhibitor (Thermo Fisher, #4374966, Waltham, MA, USA) on a T100 thermal cycler (BioRad, Mississauga, ON, Canada). For RT-qPCR, reactions were prepared using 1 μL of cDNA template (22.5 ng/μL) and 500 nM of forward and reverse primers in a final volume of 10 μL. PCR amplification was performed using an QuantStudio 3 Real-Time PCR System (Applied Biosystems, Thermo Fisher Scientific, Waltham, MA, USA) with Luna Universal Master Mix (New England Biolabs Ltd., #M3003, Whitby, ON, Canada). For normalization, the housekeeping gene, GAPDH, was used as a reference. The rat-specific primer sequences used are as follows: XBP1 forward, 5′-ACGAGAGAAAACTCATGG -3′ and reverse, 5′-ACAGGGTCCAACTTGTCC-3′, and GAPDH forward, 5′-CATCAACGACCCCTTCATTGACCTCAACTA-3′ (Invitrogen, Life Technologies, Eugene, OR, USA) and reverse, 5′-TCCACGATGCCAAAGTTGTCATGG -3′ (Invitrogen, Life Technologies, Eugene, OR, USA). The comparative threshold (CT) method was used to quantify gene expression.

### 2.6. Flow Cytometry

For cytofluorimetric analyses, 1–2 × 10^5^ cells/treatment groups were treated for 24 h and were fixed in 4% PFA for 10 min at RT. The cells were then washed twice with 1X TBS and permeabilized in absolute methanol for 10 min, and then washed once in 1X TBS buffer containing 1% FBS, 2 mM EDTA, 0.1% NaN_3_. For intercellular total JNK and phospho-JNK expression, cells were respectively incubated with Anti-JNK1 + JNK2 + JNK3 (Abcam, #ab179461, Toronto, ON, Canada) or Anti-JNK1 + JNK2 + JNK3 phospho (T183 + T183 + T221) (Abcam, #ab124956, Toronto, ON, Canada) antibodies (1:100) in TBS buffer for an hour at RT. FITC-conjugated normal rabbit IgG1 (1:250) (Invitrogen, #A21246, Life Technologies, Eugene, OR, USA) secondary antibody was used for 30 min incubation at RT in the dark and phospho and total JNK were detected independently in all treatment groups, respectively. The CytoFLEX LX Platform (Beckman Coulter Life Sciences, Mississauga, ON, Canada) was used for sample acquisition (10,000 events/sample). Data were collected and then analyzed on CytExpert software (Beckman Coulter, Brea, CA, USA).

### 2.7. Statistical Analysis

All experiments were performed in duplicate for each treatment group for a total of 5 to 6 individual biological samples. Data are expressed as means ± SE. Treatment groups were compared by one-way analysis of variance (ANOVA), and Tukey–Kramer’s test was performed to identify differences between the groups using GraphPad PRISM 7.0 software (GraphPad Software Inc., San Diego, CA, USA). *p* ≤ 0.05 was considered to be significant.

## 3. Results

### 3.1. IL-10 Improves Cardiomyocyte Viability

**Figure 1:**
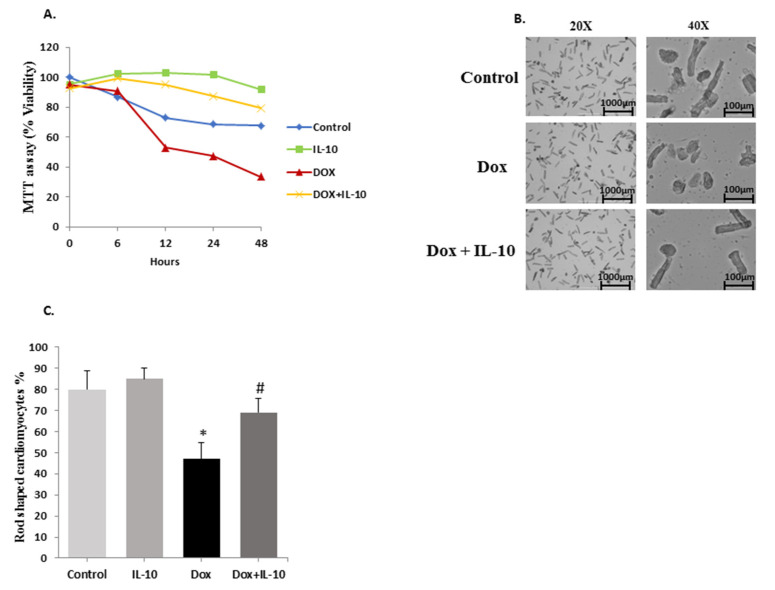
IL-10 improves cardiomyocyte viability. (**A**) Cardiomyocyte viability in different treatment groups treated with Dox (5.43 µg/mL) in the presence or absence of IL-10 (10 ng/mL) at different time points using MTT assay. (**B**) Phase contrast microscopic images of different groups after 24 h of treatment at 20X and 40X, scale bar 1000 µm and 100 µm, respectively. (**C**) The average percentage of rod-shaped cardiomyocytes at 24 h of Dox, 10 µM treatment in the presence or absence of IL-10, 10 ng/mL as % of control. Data are means +/- SE from 4 individual experiments. * *p* < 0.01 vs. control, IL-10; # *p* < 0.01 vs. Dox.

The viability of control and treated cardiomyocytes was determined at different time points (0, 6, 12, 24, 48 h) (Figure 1A) using MTT assay as well as phase contrast microscopy. In the MTT assay, viable cardiomyocytes convert the yellow colored tetrazole dye into purple formazan dye. Dox treatment caused a significant reduction in total viability to 47% at 24 h and about 30% at 48 h (*p* < 0.01). Pre and concurrent treatment of cardiomyocytes with IL-10 significantly increased the viability to 87% (*p* < 0.01) in the Dox + IL-10 group (Figure 1A). After 48 h, 75% of the control cardiomyocytes were viable and the IL-10 treated group showed a significant increase in cell viability to about 100% (*p* < 0.01 vs. control). In phase contrast microscopy, the percentage of rod-shaped cells was seemingly reduced from 84% to 45% after 24 h of Dox treatment (Figure 1B,C). IL-10 treatment in the Dox group increased the percentage of the rod-shaped cardiomyocytes significantly to 70% (Figure 1C).

### 3.2. IL-10 Effects on Dox-Induced Changes in ER-Stress Markers

**Figure 2:**
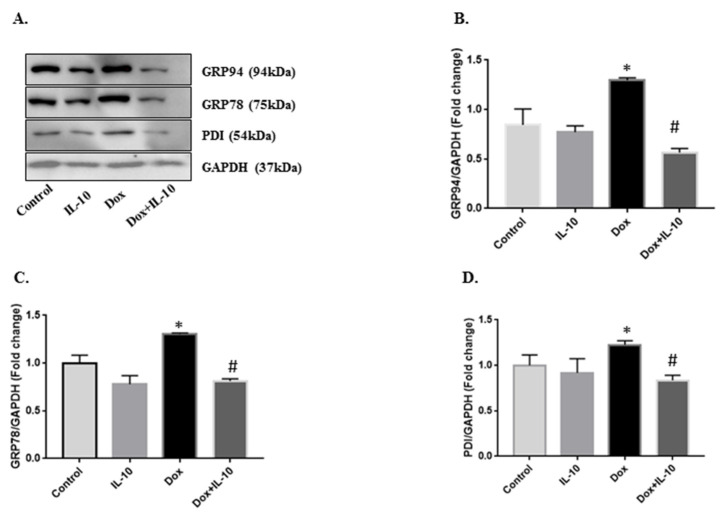
IL-10 effects on Dox-induced changes in ER stress markers. Expression of the unfolded protein response (UPR) chaperones in isolated cardiomyocytes treated with doxorubicin (Dox, 5.43 µg/mL) in the presence or absence of interleukin-10 (IL-10, 10 ng/mL) for 24 h. (**A**) Western blot of respective proteins using KDEL antibody (1:1000), which recognizes GRP94, GRP78, and PDI. Densitometric analysis of respective proteins (**B**) GRP94; (**C**) GRP78; and (**D**) PDI. GAPDH was used as the loading control. Data are means +/− SE from 5 individual experiments. * *p* < 0.001 vs. control, IL-10; # *p* < 0.001 vs. Dox.

Isolated cardiomyocytes were exposed to 5.43 µg/mL of Dox with or without IL-10 for 4, 12, and 24 h and the expression of GRP78, GRP94, and PDI proteins were recorded in different treatment groups. Expression of these three chaperone proteins was significantly increased in the Dox group at 24 h (*p* < 0.001) compared to both control and IL-10 groups (Figure 2). This increase in all the proteins were significantly (*p* < 0.001) inhibited by IL-10 treatment (Figure 2).

### 3.3. IL-10 Effect on Dox-Induced Changes in UPR-Signaling Transducers

**Figure 3:**
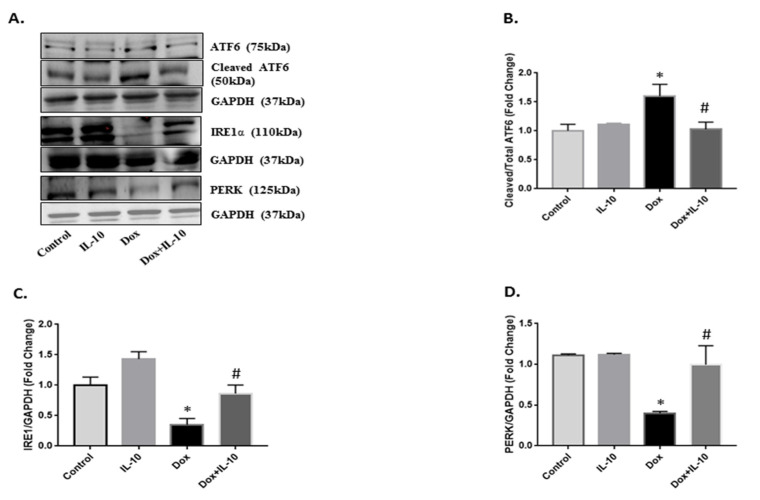
IL-10 effect on Dox-induced changes in UPR-signaling transducers. Expression of UPR-signaling transducers in isolated cardiomyocytes treated with Dox (5.43 µg/mL) in the presence or absence of IL-10 (10 ng/mL) for 24 h. (**A**) Western blot of respective proteins using activating transcription factor 6α (ATF6α) (1:1000), inositol-requiring kinase 1α (IRE1α) (1:2000), and protein kinase-like endoplasmic reticulum kinase (PERK) (1:500) antibodies. GAPDH was used as a loading control. Densitometric analysis of respective proteins (**B**) ATF6; (**C**) IRE1α; and (**D**) PERK. GAPDH was used as the loading control except for ATF6. Data are means +/− SE from 3 individual experiments. * *p* < 0.05 vs. control, IL-10; # *p* < 0.05 vs. Dox.

**Figure 4:**
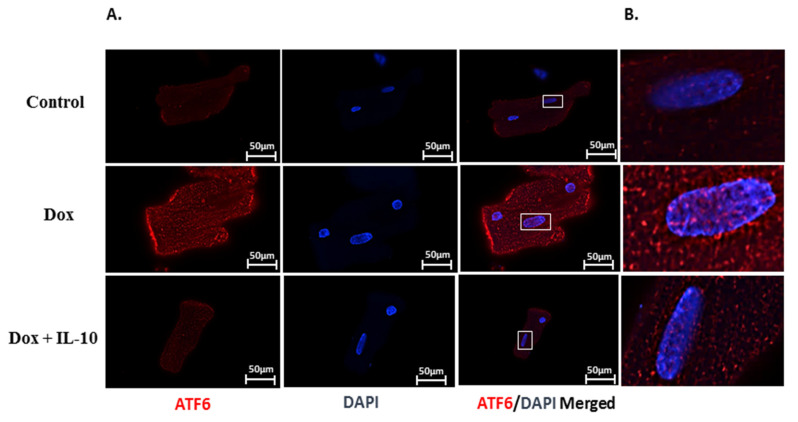
IL-10 effects on Dox-induced ATF6 cleavage and its translocation to the nucleus. Immunofluorescence images of total and cleaved activating transcription factor-6 (ATF6) in isolated cardiomyocytes treated with Dox (5.43 µg/mL) in the presence or absence of IL-10, 10 ng/mL for 24 h. (**A**) Cells were fixed and incubated with specific ATF6 antibody (1:100) overnight, followed by secondary antibody labeled with Alexa 647 (Red). DAPI (Blue) was used to locate nuclei. Final magnification is 60X in oil, scale bar 50 µm. (**B**) Enlarged images of areas marked by rectangles in ATF6/DAPI merged images highlight translocation of cleaved ATF6 in the nucleus.

We examined the protein expressions of three main ER signaling transducers (ATF6, IRE1α, and PERK). Under Dox (5.43 µg/mL) treatment for 24 h, total ATF6 as well as its activated cleaved 50 kd fragment levels were significantly (*p* < 0.05) higher compared to the control (Figure 3A,B). We also visualized the translocation of ATF6 cleaved fragment into the nucleus using immunofluorescence, as presented in the merged images (Figure 4A,B). IL-10 treatment of Dox-treated cells suppressed the total as well as cleaved ATF6 expression (*p* < 0.05) (Figure 3A,B and Figure 4A,B). Both IRE1α and PERK expressions were significantly lower on Dox treatment (*p* < 0.05) (Figure 3A,C,D). These Dox-induced changes in the levels of IRE1α and PERK were completely prevented by IL-10 and the levels were close to control levels (Figure 3C,D).

### 3.4. IL-10 Effects on Dox-Induced Changes in XBP1 mRNA and XBP1s/XBP1u Protein Levels

**Figure 5:**
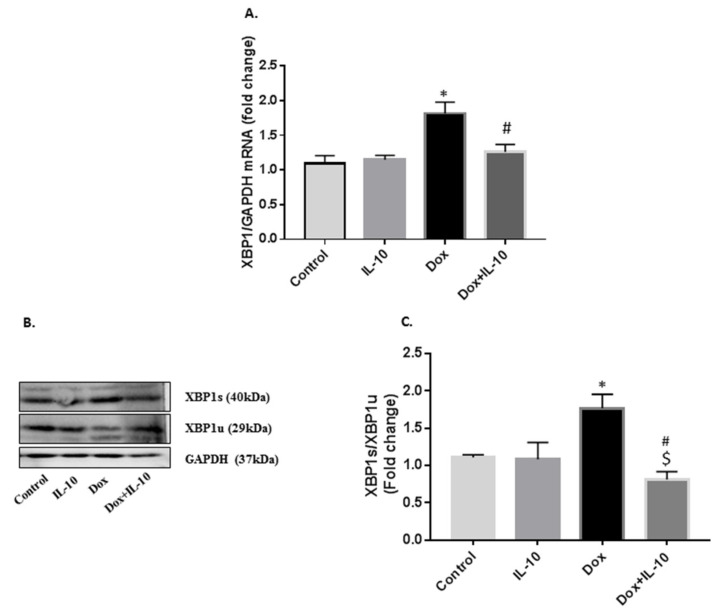
IL-10 effects on Dox-induced changes in XBP1 mRNA and XBP1s/XBP1u protein levels. Expression of X-box binding protein 1 (XBP1) in isolated cardiomyocytes treated with Dox (5.43 µg/mL) in the presence or absence of IL-10 (10 ng/mL) for 24 h. (**A**) Relative expression of mRNA levels of XBP1 using GAPDH as endogenous control. (**B**) Protein levels of spliced XBP1 (XPB1s) and unspliced XBP1 (XBP1u) using XBP1 antibody (1:500). GAPDH is shown as a loading control. (**C**) Densitometric analysis of XBP1s/XBP1u fold change. Data are means +/− SE from 5 individual experiments. * *p* < 0.005 vs. control, IL-10; # *p* < 0.005 vs. Dox; $ *p* < 0.05 vs. control.

Levels of XBP1 mRNA are critical in controlling the availability of UPR chaperones and ER-associated degradation elements. Dox caused a significant increase in XBP1 mRNA levels (Figure 5A). IL-10 was able to decrease these mRNA levels of XBP1 back to control (*p* < 0.005 vs. Dox). Dox treatment led to the splicing of XBP1 resulting in XBP1s expression (Figure 5B), which was inhibited in the Dox + IL-10 group (*p* < 0.005 vs. Dox). However, Dox decreased the levels of XBP1u, and this effect was reversed by IL-10 (Figure 5B). The ratio of XBP1s/XBP1u was increased by Dox and this effect was blunted by IL-10 (Figure 5B).

### 3.5. Effect of IL-10 on Dox-Induced Apoptotic Pathways

**Figure 6:**
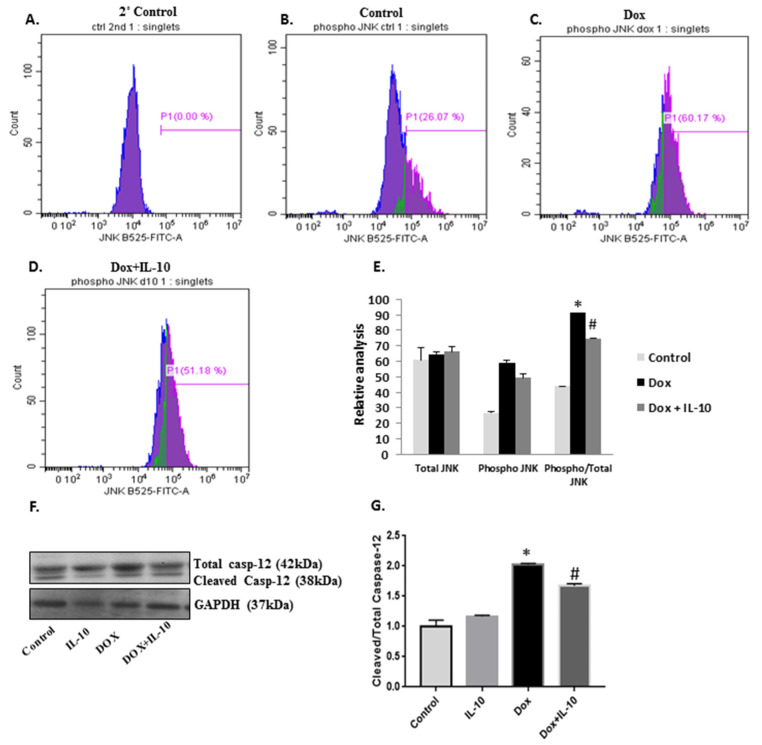
Effects of IL-10 on ER stress-induced apoptotic proteins. Expression of the ER stress-induced apoptotic proteins in isolated cardiomyocytes treated Dox (5.43 µg/mL) in the presence or absence of IL-10 (10 ng/mL) for 24 h. Flow cytometric analysis of treated cardiomyocytes; (**A**) secondary antibody control; (**B**) untreated control; (**C**) Dox; and (**D**) Dox + IL-10 of phospho- c-JUN NH2 kinase (JNK) using JNK antibody (1:100). (**E**) Relative analysis of total, phospho JNK, and phospho/total JNK in control, Dox, and Dox + IL-10 groups using flow cytometry. (**F**) Western blot of total and cleaved caspase-12 using caspase-12 antibody (1:1000). GAPDH is shown as a loading control. (**G**) Densitometric analysis of cleaved caspase-12/total caspase-12. Data are means +/− SE from 4 individual experiments. * *p* < 0.001 vs. control; # *p* < 0.005 vs. Dox.

**Figure 7:**
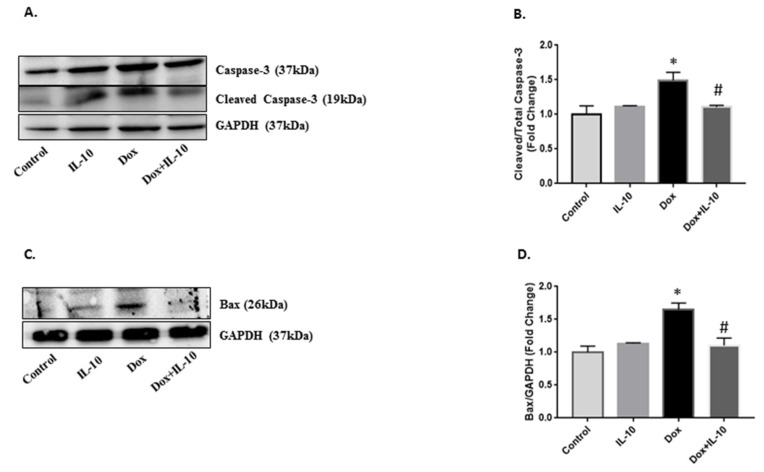
Effects of IL-10 on mitochondrial apoptotic proteins. Expression of apoptosis-inducing proteins in isolated cardiomyocytes treated with Dox (5.43 µg/mL) in the presence or absence of IL-10 (10 ng/mL) for 24 h. (**A**) Western blot of cleaved and total caspase-3 using caspase-3 antibody (1:1000); GAPDH is shown as a loading control. (**B**) Densitometric analysis of cleaved caspase-3/total caspase-3. (**C**) Western blots of Bax protein using Bax antibody (1:1000). (**D**) Densitometric analysis of Bax using GAPDH as a loading control. Data are means +/− SE from 5 individual experiments. * *p* < 0.05 vs. control, IL-10; # *p* < 0.05 vs. Dox.

ER stress is known to induce JNK phosphorylation in an IRE1α-dependent pathway to induce apoptosis when UPR fails to restore the homeostasis. We recorded a significant upregulation of JNK phosphorylation in Dox-exposed cardiomyocytes as compared to both control and IL-10 groups (*p* < 0.01). IL-10 was able to reduce the JNK phosphorylation significantly in the Dox-treated group (*p* < 0.005 vs. Dox). Caspase-12 initiates ER stress-induced apoptosis and was significantly upregulated on Dox treatment (Figure 6F,G) (*p* < 0.001 vs. control). IL-10 decreased the expression of cleaved caspase-12 significantly in the Dox + IL-10 group (*p* < 0.005 vs. Dox). Dox treatment caused a significant increase in the activation of caspase-3 in mitochondria, a downstream target of caspase-12, and this effect of Dox was also significantly blocked by IL-10 treatment in the Dox + IL-10 group (Figure 7A,B). Another mitochondrial apoptotic protein, Bax, was also significantly (*p* < 0.05) upregulated by Dox and the effect was blunted by IL-10 (Figure 7C,D).

## 4. Discussion

One of the primary aims of this study was to explore whether ER stress plays an intermediary role in Dox-induced apoptosis, and if so, which of the different ER transmembrane stress sensors is more activated. MTT assay data, corroborated by our phase contrast studies, provided some strong evidence that Dox causes cardiomyocyte cell death in a time-dependent manner. Similar findings of Dox-induced cardiomyopathy have been reported before by us [5,7] and by many others [32,33,34]. However, it is important to note that in the present study this cell kill was almost completely prevented by IL-10 in the Dox + IL-10 group. The latter showed significantly higher viability and preserved rod-shaped morphology of the isolated cardiomyocytes, compared to the Dox group (Figure 1). IL-10 is reported to have anti-inflammatory [35,36,37], antioxidant [28], anti-fibrotic [27,38], and pro-angiogenesis [39] effects in the heart. IL-10 has also been shown to improve cardiac function under different stress conditions [28,30,31]. These and other data in the present study provided strong evidence for a potential role of IL-10 in mitigating Dox-induced ER stress as well as cardiomyocyte apoptosis.

In this regard, we also noted that Dox caused an expression of GRP78, GRP94, and PDI (Figure 2), which are often used as stress markers [40] and are important in protein folding as well as degradation of misfolded proteins to maintain ER homeostasis [13]. Furthermore, overexpression of these ER stress markers can be stimulus for the activation of one or more of the UPR transducers, such as ATF6, IRE1α, and/or PERK [41,42]. We found that in Dox treatment, ATF6 was overexpressed in the cells and cleaved ATF6 was also expressed in the cells and appeared in the nucleus (Figure 4). Activated ATF6 induces the transcription of downstream molecules such as XBP1, which was also upregulated on Dox treatment (Figure 5). These ER sensor responses were blocked by IL-10. The inhibition of ATF6/XBP1 response by IL-10 restored ER homeostasis and reduced cell death. These data also support the argument that Dox-induced apoptosis is mediated by ER stress.

Interestingly, both IRE1α and PERK expressions were suppressed by Dox (Figure 3). IRE1α is important for the splicing of XBP1 mRNA to produce XBP1s and XBP1u. XBP1s is responsible for the transcription of anti-oxidant and anti-inflammatory genes in the nucleus [19] and as XBP1s expression was not completely blocked in the control and IL-10 groups, the data may suggest its useful role in ER homeostasis. However, a significant increase in the expression of the XBP1 gene, as well as its spliced protein on Dox treatment in the absence of IRE1α, suggests that IRE1α may not be solely responsible for XBP1 splicing but rather ATF6 activation may have played an important role in the XBP1 splicing process, which is yet to be investigated. Another interesting finding on prior treatment of IL-10 in Dox-treated cardiomyocytes was its ability to induce expression of XBP1u, a negative regulator of XBP1s. Zhao and associates [43] have reported the importance of XBP1u in maintaining the vascular smooth muscle cell’s contractile phenotype and the suppression of vascular inflammation and proteolytic degradation. XBP1u can form a complex with XBP1s and repress transcription of iNOS [44]. XBP1u has emerged as a critical player in accurate termination of UPR by enhancing degradation and nuclear exclusion of XBP1s during the post ER stress recovery phase [45]. XBP1u also acted as a regulator of IRE1α in that its suppression was reversed by IL-10 treatment in the Dox + IL-10 group. Thus, overexpression of IRE1α and XBP1u in the Dox + IL-10 group helped reduce the overall ER stress induced by Dox and may have helped in the recovery of ER homeostasis and cell survival.

Caspase-12 is an ER resident proapoptotic protein, which upon cleavage promotes apoptosis [22,23,24,25,46]. Upon Dox treatment, we saw activation of caspase-12 as well as JNK, further confirming the involvement of ER stress-initiated apoptosis (Figure 6). IL-10 was able to reduce overexpression of both procaspase-12 and cleaved caspase-12 by reducing overall ER stress and promoted cell survival. The IRE1α arm of UPR can also initiate JNK phosphorylation to stimulate the ER stress-induced apoptotic pathway [18]. However, Dox downregulated IRE1α, suggesting JNK phosphorylation can occur in an ER-independent pathway as well. Dox is known to upregulate pro-inflammatory cytokine interleukin-1 beta (IL-1β), which can also promote JNK phosphorylation, and thus cell apoptosis [47,48]. Previous studies have shown that IL-10 has the ability to induce shedding of the IL-1 type I receptor, which can reverse the IL-1β-induced stimulation of JNK activity, and thus promotes cellular homeostasis [49]. In our study, Dox also increased Bax protein expression which leads to an activation of caspase-3 and initiation of the apoptosis through mitochondrial stress. IL-10 treatment blocked the involvement of mitochondrial stress in Dox-treated cardiomyocytes by inhibiting Bax and cleaved caspase-3 expression, and thus promoted cell survival (Figure 7).

It is also known that IL-10 signaling is through the phosphorylation of the cytoplasmic tails of its receptor 1 and receptor 2 by JAK1 and Tyk2, respectively [35,50]. Ultimately, phosphorylated STAT3 homodimer translocates to the nucleus to activate the expression of target genes that largely define the anti-inflammatory response of IL-10. Furthermore, the activation of ER stress itself can inhibit STAT3 phosphorylation, which may suppress IL-10 effects [51]. It is also reported that IL-10 induces apoptosis of regulatory T-cells [52]. It seems that molecular mechanisms that govern IL-10 production and action may be governed by unique conditions in different cell pathologies and cell type. Our work shows that IL-10 inhibits Dox-induced ER stress through several potential sites including dissociation of GRP78 from any of its downstream effector proteins for its action.

## 5. Conclusions

**Figure 8:**
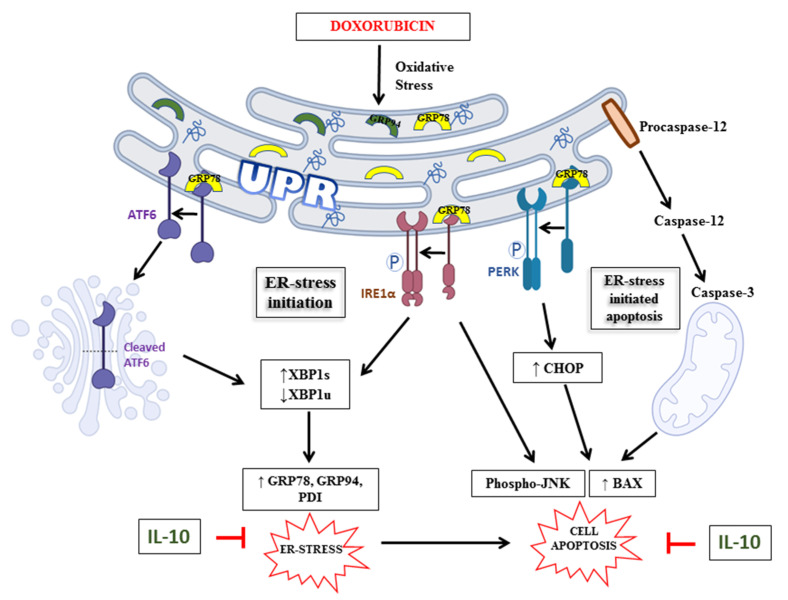
An overview of Dox-induced ER stress and apoptosis as well as its mitigation by IL-10. Dox-induced oxidative stress can disturb the ER homeostasis leading to the accumulation of unfolded and/or misfolded proteins. This unfolded protein response (UPR) then leads to the activation of UPR transducers: ATF6, IRE1α, and PERK via dissociation of GRP78. ATF6 cleavage in the Golgi body increases XBP1s and inhibits XBP1u, followed by an upregulation of ER stress markers (GRP78, GRP94, and PDI). Dox also initiates an activation of procaspase-12, which is known to activate caspase-3, BAX, and apoptosis through mitochondria. The exogenous administration of IL-10, one hour prior to the exposure of cardiomyocytes to Dox for 24 h, was found to be effective in suppressing the expressions of ER stress markers (GRP78, GRP94, PDI, and XBP1), as well as reducing the activation of procaspase-12, JNK phosphorylation, Bax activation, and cell death. ↑: upregulation; ↓: downregulation.

In conclusion, our findings demonstrate that Dox significantly induces an overexpression of ER stress-related proteins in isolated cardiomyocytes and decreases their viability in a time-dependent manner (Figure 8). The beneficial effects of IL-10 in modulating ER stress and ER-initiated apoptosis (Figure 8) may prove to be a significant advance in restricting cardiac damage during stress or inflammation. The recognition of Dox-induced cardiotoxicity as well as a dire need for a cardioprotective agent to be administered in conjunction with Dox is evident, and IL-10 may prove to be such an agent.

## Data Availability

Data are contained within the article. Original raw data on gels are appended.
